# Capturing *Salmonella* SspH2 Host Targets in Virus-Like Particles

**DOI:** 10.3389/fmed.2021.725072

**Published:** 2021-09-08

**Authors:** Margaux De Meyer, Igor Fijalkowski, Veronique Jonckheere, Delphine De Sutter, Sven Eyckerman, Petra Van Damme

**Affiliations:** ^1^iRIP Unit, Department of Biochemistry and Microbiology, Ghent University, Ghent, Belgium; ^2^Vlaams Instituut voor Biotechnologie Center for Medical Biotechnology, Vlaams Instituut voor Biotechnologie, Ghent, Belgium; ^3^Department of Biomolecular Medicine, Ghent University, Ghent, Belgium

**Keywords:** effectors, interactomics, infection biology, *Salmonella*, SspH2, type III secretion, virotrap

## Abstract

In the context of host-pathogen interactions, gram-negative bacterial virulence factors, such as effectors, may be transferred from bacterial to eukaryotic host cytoplasm by multicomponent Type III protein secretion systems (T3SSs). Central to *Salmonella enterica* serovar Typhimurium (*S*. Typhimurium) pathogenesis is the secretion of over 40 effectors by two T3SSs encoded within pathogenicity islands SPI-1 and SPI-2. These effectors manipulate miscellaneous host cellular processes, such as cytoskeleton organization and immune signaling pathways, thereby permitting host colonization and bacterial dissemination. Recent research on effector biology provided mechanistic insights for some effectors. However, for many effectors, clearly defined roles and host target repertoires—further clarifying effector interconnectivity and virulence networks—are yet to be uncovered. Here we demonstrate the utility of the recently described viral-like particle trapping technology Virotrap as an effective approach to catalog *S*. Typhimurium effector-host protein complexes (EH-PCs). Mass spectrometry-based Virotrap analysis of the novel E3 ubiquitin ligase SspH2 previously shown to be implicated in modulating actin dynamics and immune signaling, exposed known host interactors PFN1 and−2 besides several putative novel, interconnected host targets. Network analysis revealed an actin (-binding) cluster among the significantly enriched hits for SspH2, consistent with the known localization of the *S*-palmitoylated effector with actin cytoskeleton components in the host. We show that Virotrap complements the current state-of-the-art toolkit to study protein complexes and represents a valuable means to screen for effector host targets in a high-throughput manner, thereby bridging the knowledge gap between effector-host interplay and pathogenesis.

## Introduction

*Salmonella* is a gram-negative genus of intracellular pathogenic bacteria, which may cause divergent disease outcomes ranging from gastroenteritis to enteric and typhoid fever. Virulence of salmonellae relies on the engagement of two Type III secretions systems (T3SS-1 and−2) encoded within *Salmonella* pathogenicity island (SPI)-1 and −2, respectively (1). The proteinaceous T3SSs serve as a conduit for translocation of effector proteins from bacterial to host cytoplasm. Broadly speaking, effectors transported by T3SS-1 facilitate invasion of epithelial cells, while T3SS-2 effectors are vital for intracellular persistence and systemic infection (2). Effector-mediated invasion includes induction of a phagocytosis-like process, resulting in bacterial enclosure in an endosomal compartment—termed the *Salmonella*-containing vacuole (SCV) (3). An estimated total of 28 T3SS-2 effectors are believed to be translocated across the vacuolar membrane into the host cytoplasm by *Salmonella enterica* serovar Typhimurium (*S*. Typhimurium), acting to render an intracellular replicative niche for the pathogen (4). Among this series of effectors are three members of the NEL (novel E3 ligase) family ubiquitin (Ub) ligases: SspH1 (*Salmonella* secreted protein H1), SspH2 and SlrP (*Salmonella* leucine-rich repeat protein) (5). SlrP and SspH2 are found in most *Salmonella enterica* strains, while SspH1 (69% identity to SspH2) occurrence is restricted to certain serovar *S*. Typhimurium strains (e.g., strain 14,028 s) (6). Moreover, SspH2 is presumed to be uniquely translocated by T3SS-2, which is in contrast to the other two *S*. Typhimurium NEL family members. These three ubiquitin ligases possess an N-terminal leucine rich repeat (LRR), a motif frequently involved in the formation of protein/protein interactions (PPIs), that shields the unique C-terminal NEL catalytic domain. This autoinhibitory conformation is presumed to be lifted upon substrate binding (7). Host substrates that effectuate different host cell signaling pathways have been identified for each of the *S*. Typhimurium NEL family members (8–10). More specifically, ubiquitination of protein kinase-1 (PKN1) by SspH1 was shown to be implicated in inhibiting NF-κβ signaling and reduced Interleukin-8 (IL-8) secretion (8, 11). SspH2-mediated ubiquitination of NOD1 on the other hand was shown to enhance IL-8 secretion in infected cells (9). Thioredoxin (TRX) was identified as a host substrate of SlrP, leading to increased cell death upon ubiquitination (10). Moreover, Miao and colleagues reported a contribution of SspH1 and SspH2 to *S*. Typhimurium bovine virulence and a role for SlrP in systemic murine infection (6). In addition, SspH2 was shown to act as an anti-inflammatory effector in *S*. Enteridis, showing increased inflammatory cytokine production and decreased IL-8 synthesis when using *sspH2* deletion mutants in infected human intestinal cells and organ homogenates (12).

**Figure 1:**
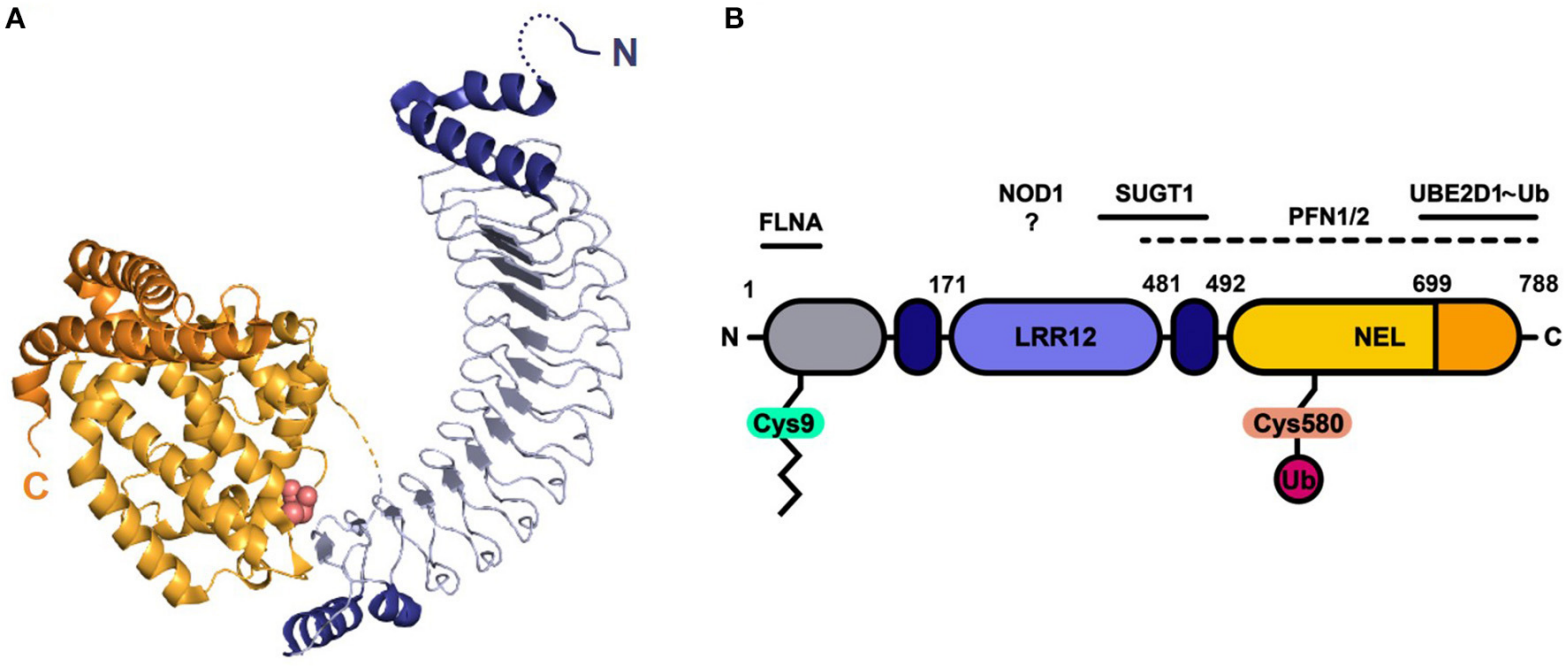
Protein structure and domain architecture of *S*. Typhimurium effector SspH2. **(A)** The ribbon diagram represents the by X-ray crystallography solved protein structure of SspH2 residues 166-783 (PDB 3G06), consisting of two main domains: the leucine-rich repeat (LRR) domain (blue) and the novel E3 ligase (NEL) domain (orange). Both sides of the 12 LRRs are capped with alpha helices (dark blue). The NEL domain harbors the catalytic Cys residue (pink atoms shown in spheres) and can be further subdivided into a globular domain (light orange) and a 2-helical C-terminal extension (dark orange). **(B)** A linear representation of the SspH2 architecture and its reported host interactors ubiquitin conjugating enzyme E2 D1 (UBE2D1), suppressor of G2 allele of SKP1 homolog (SUGT1), nucleotide binding oligomerization domain containing 1 (NOD1), profilin 1 (PFN1) and 2 (PFN2) and filamin-A (FLNA). SspH2 is *S*-palmitoylated (zigzag line) at Cys9, attaching the effector to the host membrane. As an E3 ubiquitin (Ub) ligase, catalytic Cys580 of SspH2 is involved in the transfer of Ub to substrate proteins. Color-coding of domains is done according to panel **(A)**. Gray region represents sequence not covered in the crystal structure.

Like its NEL family members, SspH2 is composed of two main domains, i.e., an LRR and NEL domain (Figure 1) (7). Capped at the N- and C-terminal sites by α-helices, the SspH2 LRR domain consisting of 12 repeats constitutes SspH2 residues 171-481. A loop of 10 residues connects the LRR to the all-helical NEL domain that holds the catalytical Cys residue and comprises a globular region (residues 491–699) followed by two interacting α-helices. *In vitro*, binding of SspH2 to the Ub-conjugated E2 ligase Ub-conjugating enzyme E2 D1 (UBE2D1) via its NEL domain was reported to generate Lys48-linked polyubiquitin chains (13). Conversely, Bhavsar et al. identified monoubiquitination of NOD1 by SspH2 in HeLa cells as a putative prerequisite of modulating nucleotide-binding leucine rich repeat receptor (NLR) signaling (9). Interestingly, binding of NLR co-chaperone SUGT1 (Suppressor of G2 allele of SKP1 homolog 1) to SspH2 was vital for the observed immune response upon NOD1 ubiquitination (9). Interaction between SUGT1 and SspH2 was identified using quantitative proteomics by means of AP-MS and stable isotope labeling by amino acids (SILAC) in human embryonic kidney 293T (HEK293T) cells and verified using reciprocal co-immunoprecipitation (co-IP) (14). This interaction was later on confirmed using a similar strategy in HeLa cells (15). Next to interactors implicated in NLR signaling, SspH2 has been extensively studied in the context of actin cytoskeleton dynamics with reported interactions of the effector with profilin-1 (PFN1), PFN2 and filamin-A (FLNA) in infected cells (15, 16), proteins implicated in polymerization and branching of actin filaments, respectively. In addition, by itself, SspH2 was also observed to decrease actin polymerization *in vitro* (16). In addition to its substrate NOD1, several other host interactors have been identified for the SspH2 NEL ubiquitin ligase. Further, an ubiquitinome screen revealed SspH2-regulated diGly sites among others within SUGT1, PFN2 and FLNA when comparing the ubiquitinomes of wild-type (WT) and Δ*sspH2 S*. Typhimurium-infected HeLa cells (15).

Analogous to *S*. Typhimurium effector SseI (*Salmonella* secreted effect I), SspH2 is known to be *S*-palmitoylated within the N-terminus (Cys9), a modification linked with its plasma membrane localization in (infected) cells (17). However, SspH2 and SseI exhibit differential membrane localization, suggesting additional determinants for localization apart from their shared lipid modification. Interestingly, SspH2 has been shown to co-localize with vacuole-associated actin polymerizations (VAP) near intracellular bacterial clusters in fibroblast and macrophage-like cell lines among others (16). Based on co-localization studies, it was postulated that the interaction of SspH2 with filamin mediates the specific cytoskeletal localization at the plasma membrane to regions of dynamic actin polymerization (16). Although various cytoskeletal host proteins have been shown to (directly) associate with SspH2, the mode of effector functioning and contribution to virulence in animal infection models still remains largely elusive.

Recent large-scale efforts to map *S*. Typhimurium effector-host protein complexes (EH-PCs) include a complementary proximity-dependent biotin identification (BioID) and AP-MS effort (18), while crosslinking affinity purification mass spectrometry (AP-MS) of chromosomally encoded affinity-tagged effector proteins delivered upon host infection was used by Walch and colleagues (19). BioID relies on a promiscuous biotin ligase fused to the protein under study, or in this case effector protein, to biotinylate accessible Lys residues of proximate or interacting proteins (20). Benefiting from the covalent biotin label permitting stringent washing steps, BioID proved to be adept at identifying (integral) membrane proteins and poorly soluble proteins, like cytoskeleton components, in the reported *S*. Typhimurium BioID dataset (18). In contrast, classical AP-MS relies on co-purification and preservation of interactions upon lysis conditions, making the isolation of membrane (-associated) and less soluble proteins more challenging. In the Walch study, SspH2 was examined using AP-MS with crosslinking, revealing potential interactors upon native delivery of the tagged effector in macrophage-like cells, such as myosin heavy chain 9 (MYH9), also previously identified in HeLa cells (15), along with 4 other myosin proteins, i.e., MYO1E, MYL6, MYO1F and MYO5A.

**Figure 2:**
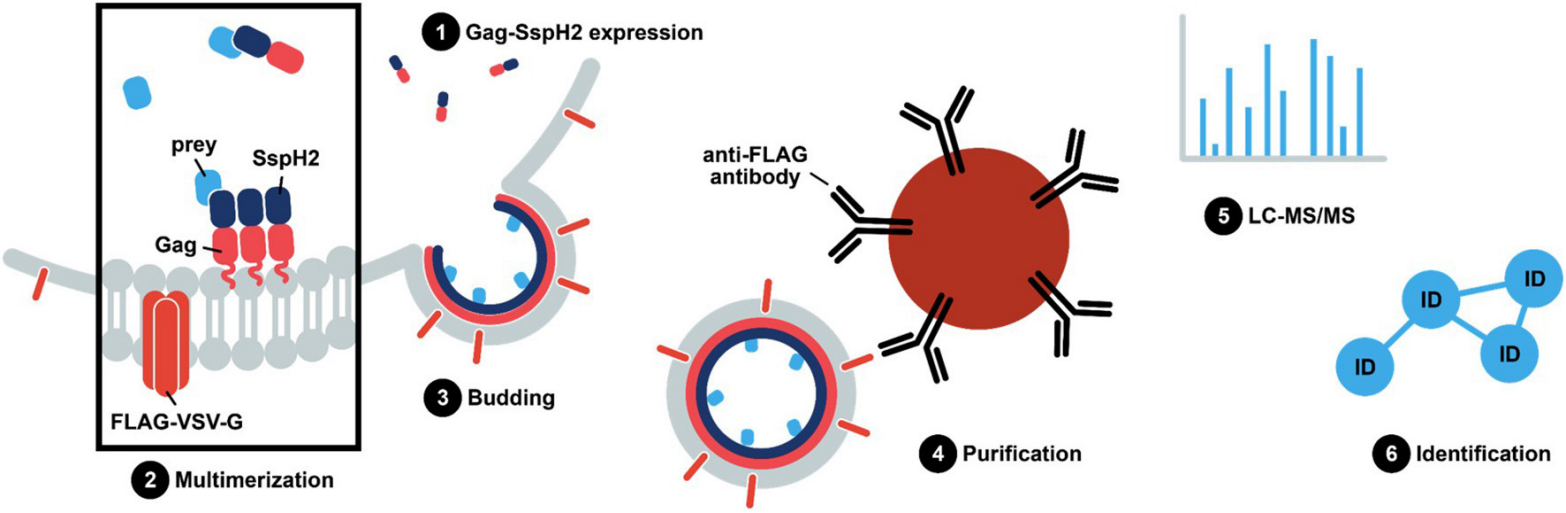
*S*. Typhimurium SspH2 protein complex purification using Virotrap. In Virotrap, a fusion of the bait, e.g., effector protein of interest, to the C-terminus of the Gag polyprotein (myristoylated; pink zigzag line) of the human immunodeficiency virus type 1 (HIV-1) is transiently expressed in HEK293T cells. Along with the Gag-bait fusion, FLAG-tagged vesicular stomatitis virus glycoprotein (VSV-G) is expressed and becomes embedded in the plasma membrane. Gag facilitates multimerization and localization of the fusion protein at the plasma membrane, giving rise to virus-like particle (VLP) budding. Consequently, host preys are trapped inside VLPs that can be captured in the cell medium by anti-FLAG immunoprecipitation. LC-MS/MS-based analysis of the VLP content subsequently allows identification of significant bait co-enriched host proteins.

**Figure 3:**
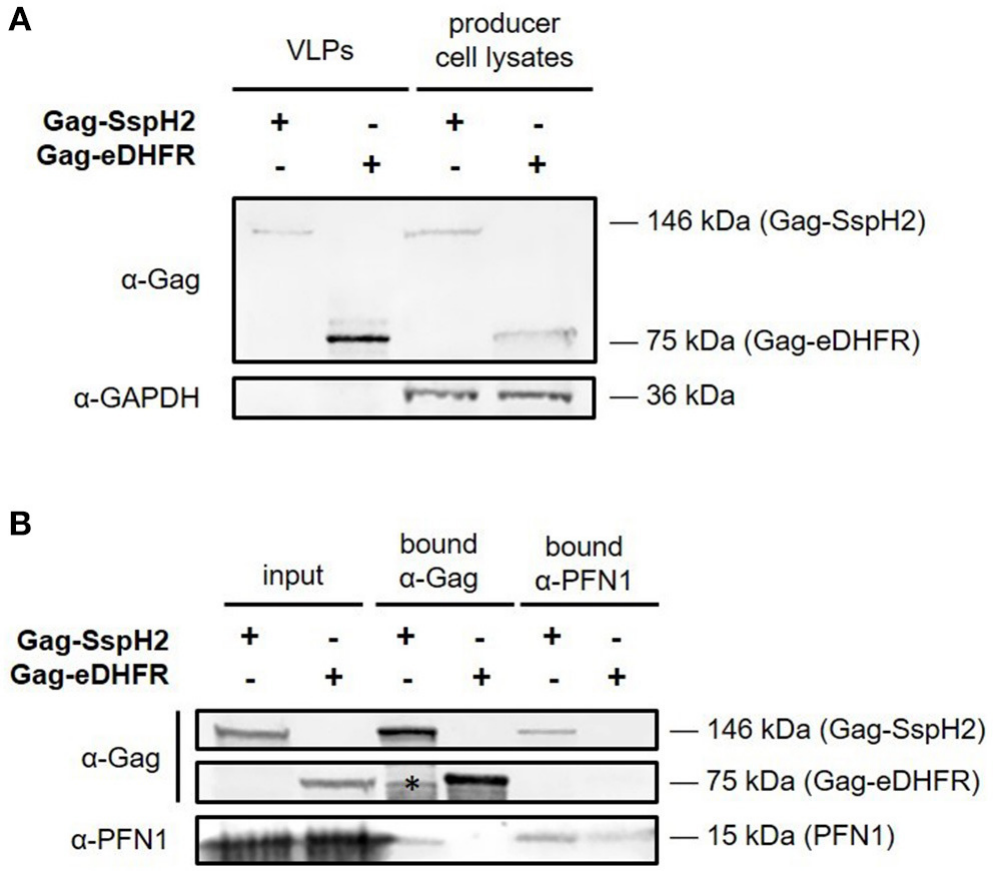
Western blot analysis of Gag-SspH2 virus-like particles and interaction with profilin. Virotrap control Gag-eDHFR and *S*. Typhimurium effector Gag-SspH2 fusion was co-expressed with FLAG-tagged vesicular stomatitis virus glycoprotein (VSV-G) in HEK293T cells. **(A)** For both setups, virus-like particles (VLPs) were captured from the cell medium using anti-FLAG immunoprecipitation. VLP lysates loaded on gel represent a 20-fold equivalent of the producer cell lysate loaded. **(B)** Reciprocal co-immunoprecipitation was done with producer cell lysates of cells expressing Gag-eDHFR and Gag-SspH2 using anti-Gag and anti-PFN1 antibodies. The asterisk indicates a non-specific/degradation signal.

The virus-like particle trapping technology—Virotrap—includes generation of a translational fusion protein with the protein of interest to the C-terminus of the human immunodeficiency virus 1 (HIV-1) Gag protein (Figure 2). Gag elicits vesicle budding containing the Gag-effector fusion upon expression in HEK293T cells (Figure 3). Consequently, protein complexes are trapped inside virus-like particles (VLPs). VLPs can subsequently be purified from the cell medium, thereby circumventing the need for cell lysis. Here, we describe Virotrap as a method to study EH-PCs, using *S*. Typhimurium SspH2 as a model effector. We verify previously described host targets and identify several novel potential host interactors. Overall, we show that Virotrap is adept at identifying host targets of *Salmonella* effector protein SspH2, demonstrating its applicability to complement the general state-of-the-art toolkit to study EH-PCs.

## Materials and Methods

### Cell Culture

HEK293T cells obtained from (21) were maintained in high-glucose Dulbecco's Modified Eagle Medium containing GlutaMAX (DMEM; Gibco, cat no. 10566016), supplemented with 10% fetal bovine serum (FBS, Gibco, cat no. 10270106) and 100 U/mL penicillin/streptomycin (Gibco, cat no. 15070063) in a humidified incubator at 37°C and 5% CO_2_.

### Bacterial Strains

All cloning steps were performed in *Escherichia coli* strain DH10B using standard chemical transformation. The *S. enterica* serovar Typhimurium (*S*. Typhimurium) wild-type strain SL1344 (Genotype: hisG46, Phenotype: His (-); biotype 26i) used for genomic DNA extraction and *sspH2* amplification was obtained from the *Salmonella* Genetic Stock Center (SGSC, Calgary, Canada; cat no 438).

### Genomic DNA Extraction

A liquid stationary *S*. Typhimurium culture grown in Luria broth, Miler formulation (10 g/L Tryptone, 5 g/L yeast extract, 10 g/L NaCl) was pelleted and lysed overnight at 37°C in lysis buffer (20 mM Tris-HCl pH 7.5, 5 mM EDTA, 150 mM NaCl, 0.15 mg/mL proteinase K and 0.2% SDS). The bacterial lysate was separated using a phase lock gel (phase lock gel heavy, 5PRIME, Quantabio) and Phenol:Chloroform:Isoamyl Alcohol (25:24:1, v/v) extraction. The DNA was precipitated from the aqueous phase using a final concentration of 50% isopropanol and 0.3 M NaCl and the pellet was washed in 70% ethanol and resuspended in 10 mM Tris-HCl pH 8 and 1 mM EDTA.

### Plasmids

*S*. Typhimurium sspH2 was PCR amplified from genomic SL1344 DNA using the primers GGGGACAAGTTTGTACAAAAAAGCAGGCTTAATGCCCTTTCATATTGGAAGC and GGGGACCACTTTGTACAAGAAAGCTGGGTTCAGTTACGACGCCACTGAACG. The *sspH2* amplicon was purified by means of a Nucleospin® Gel and PCR clean-up kit (Macherey-Nagel) according to the manufacturers' instruction and cloned into the Gateway® pDONR221 and pMET7-GAG-SP1 (22) using BP Clonase II Enzyme (Invitrogen) and LR Clonase II Plus Enzyme (Invitrogen), respectively. The pMD2.G (VSV-G envelope-expressing plasmid; Addgene, plasmid no. 12259) and pcDNA3-FLAG-VSV-G (Addgene, plasmid no. 80606) plasmids were retrieved from Addgene and the pSVsport vector was obtained from Life Technologies. The correctness of the *sspH2* insert was confirmed by Sanger sequencing (Eurofins).

### SDS-PAGE and Immunoblotting

VLP and producer cell lysates were added sample loading buffer (XT sample buffer, Bio-Rad) and reducing agent (XT reducing agent, Bio-Rad) according to the manufacturer's instructions. Proteins were separated on a 4–12% gradient XT precast Criterion gel using XT-MOPS buffer (Bio-Rad) at 150 V and subsequently transferred onto a PVDF membrane. Membranes were blocked for 30 min in a 1:1 Tris-buffered saline (TBS)/Odyssey blocking solution (cat no. 927-40003, LI-COR) and probed using primary antibodies (1/1000 dilution, rabbit anti-PFN1, Abcam, cat no. 124904; 1/5000 dilution, mouse anti-Gag Abcam, cat no. 9071; 1/10000 dilution, mouse anti-GAPDH, Abcam, cat no. 8245) in TBS-T/Odyssey blocking buffer. After three washes of 10 min in TBS-T (0.1% Tween-20), membranes were incubated with secondary antibody (1/5000 dilution; IRDye 680 anti-rabbit, cat no. 926- 68071 and IRDye 800 anti-mouse, cat no. 926-32210; LI-COR) for 30 min in TBS-T/Odyssey blocking buffer. Following three washes in TBS-T and one additional wash in TBS, fluorescent detection was done using an Odyssey infrared imaging system (Odyssey Fc, LI-COR).

### Virotrap and LC-MS/MS

Virotrap was essentially performed as described previously (23). In brief, 10 million HEK293T cells were seeded per 75 cm^2^ flask (T75) in complete DMEM and transfected in duplicate the next day using polyethylenimine (PEI) reagent (linear 25 kDa, Polysciences, Inc.). The transfected DNA/PEI mixture consisted of 0.71 μg pcDNA3-FLAG-VSV-G, 0.36 μg pMD2.G, 6.43 μg pMET7-GAG-SP1-*sspH2* (bait samples) or 3.75 μg pMET7-GAG-SP1-eDHFR and 2.67 μg pSVsport (control samples) and 37.5 μL PEI (1 mg/mL solution in MQ, pH 7.0) per T75. The cellular supernatant was harvested 48 h after transfection, spun at 1,500 xg for 3 min at room temperature and filtered through a 0.45 μm Millex® filter (Millipore). Per sample (equivalent of T75), 20 μL MyOne Streptavidin T1 beads (10 mg/mL; Invitrogen), washed in 20 mM TRIS HCl pH 7.5 and 150 mM NaCl, was loaded with 2 μL anti-FLAG BioM2-biotin antibodies (1 mg/mL; ANTI-FLAG® BioM2, cat no. F9291, Sigma Aldrich) in 200 μL washing buffer by end-over-end rotation and incubation for 2 h. VLPs were allowed to bind the anti-FLAG-coated beads for 2 h by end-over-end rotation at room temperature. Bead-bound VLP complexes were washed once with washing buffer and eluted using 20 μL elution buffer (20 mM TRIS HCl pH 7.5, 150 mM NaCl, 200 μg/ml FLAG-peptide) and incubate for 30 min at 37°C. Subsequently, VLPs were lysed by addition of 2.2 μL amphipathic polymer solution (Amphipol A8-35, Anatrace; final concentration of 1 mg/mL) and incubation for 10 min. For protein concentration, proteins were pelleted from the lysates by acidification (0.2% final concentration formic acid) (24). After acidification, protein pellets were dissolved in 20 μL 50 mM triethylammonium bicarbonate (TEAB) buffer (pH 8.5), boiled and digested overnight using 0.5 μg of sequence-grade modified trypsin (Promega). After a final acidification step (0.4% formic acid, final concentration), samples were separated on an UltiMate™ 3000 RSLCnano (Thermo Scientific) and analyzed on a Q Exactive HF instrument (Thermo Scientific; 7.5 μL injected, 1.5 h long run) as described previously (25, 26).

### Co-immunoprecipitation

Virotrap particle producer cells (see previous section) were lysed in 1 mL radioimmunoprecipitation assay (RIPA) buffer (50 mM TRIS HCl pH 8, 200 mM NaCl, 2 mM Na_2_EDTA, 1% Nonidet P40, 0.5% DOC, 0.05% SDS) on ice and the lysates centrifuged for 15 min at 16,000 xg. 500 μl of cleared cell lysate was incubated with 40 μL protein G Dynabeads (Invitrogen, cat no. 10003D) pre-bound with either 2 μg anti-Gag antibodies or anti-PFN1 antibodies in 500 μL PBS (pH 7.4; Gibco, cat no. 10010023) supplemented with 0.02% Tween-20 (PBS-T). Unbound antibodies were removed by washing the beads with 500 μL PBS-T prior to addition of the lysate. Following overnight incubation at 4°C, two PBS-T washes were followed by one TBS wash. Elution of the immunoprecipitated proteins was done using 40 μL elution buffer consisting of 70% MQ, 25% XT sample buffer (Bio-Rad) and 5% XT reducing agent (Bio-Rad) at 95°C for 10 min.

### Data Analysis

Searches were performed using MaxQuant (Version 1.6.6.0) (27) against the human SwissProt Proteome Database (Release 2020-06) complemented with eDHFR, FLAG-VSV-G, VSV-G, Gag and SL1344 SspH2 protein sequences. In MaxQuant, multiplicity was set to one, indicating that no labels were used. Furthermore, we performed label-free quantification (LFQ) using MaxQuant's standard settings with a minimum of two ratio counts and only considering unique peptides for protein quantification. A decoy database of reversed protein sequences was used to estimate FDR, and 1% FDR threshold was applied. Matching between runs was implemented with a match time window of 0.7 min and an alignment time window of 20 min. Methionine oxidation and N-terminal protein acetylation were set as variable modifications and trypsin/P was set as the digestion enzyme allowing one missed cleavage. N-terminal acetylation was included in protein quantification. The MaxQuant ProteinGroups data file was processed using R Studio (R Foundation for Statistical Computing, V1.3.959) and custom R scripts. Proteins exclusively identified in the Virotrap control samples were excluded from further downstream processing and statistical analysis. The dataset was further filtered based on reversed hits, potential contaminants and proteins only identified by site. For identifications with LFQ values calculated for both Gag-SspH2 bait replicates (i.e., 2 valid values), LFQ intensities were log2 transformed and missing values were imputed using the QRILC function with default parameters from the imputeLCMD package in R (28). Significance was assessed using Limma in R (29) as previously demonstrated (30). Replicate samples were grouped and compared to the control samples (Gag-eDHFR) in a pairwise analysis. Basic data handling and Pearson correlation calculations were performed in Perseus V1.6.6.0 (31). Network analysis was done using the STRING database (32) and the open-source software Cytoscape (33) for visualization.

## Results

### Interactomics Profiling of SspH2 Host Interactors Using Virotrap

**Figure 4:**
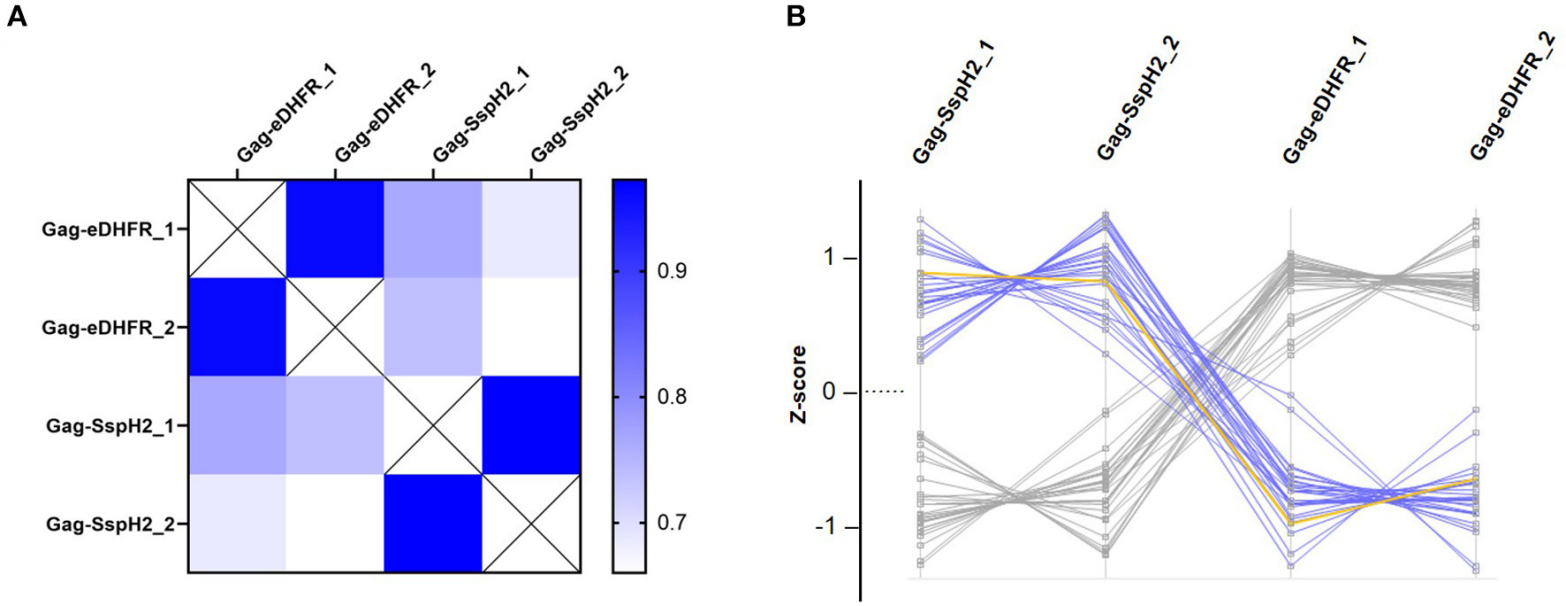
Quality control and data exploration of Virotrap samples. **(A)** Heat map visualization of pairwise LFQ Pearson correlations were calculated for duplicate (annotated “_1” and “_2”) Virotrap control (Gag-eDHFR) and effector (Gag-SspH2) samples. **(B)** Profile plots of significantly enriched protein identifications are shown after z-scoring of corresponding (imputed) LFQ intensities in the Virotrap samples. Proteins uniquely identified in the Virotrap control samples were excluded from the statistical analysis and are not shown in the profile plot. Blue highlighted lines represent significant hits co-enriched with Gag-SspH2 (yellow line) vs. the Virotrap Gag-eDHFR control setup (Limma, *p*-value < 0.05).

**Figure 5:**
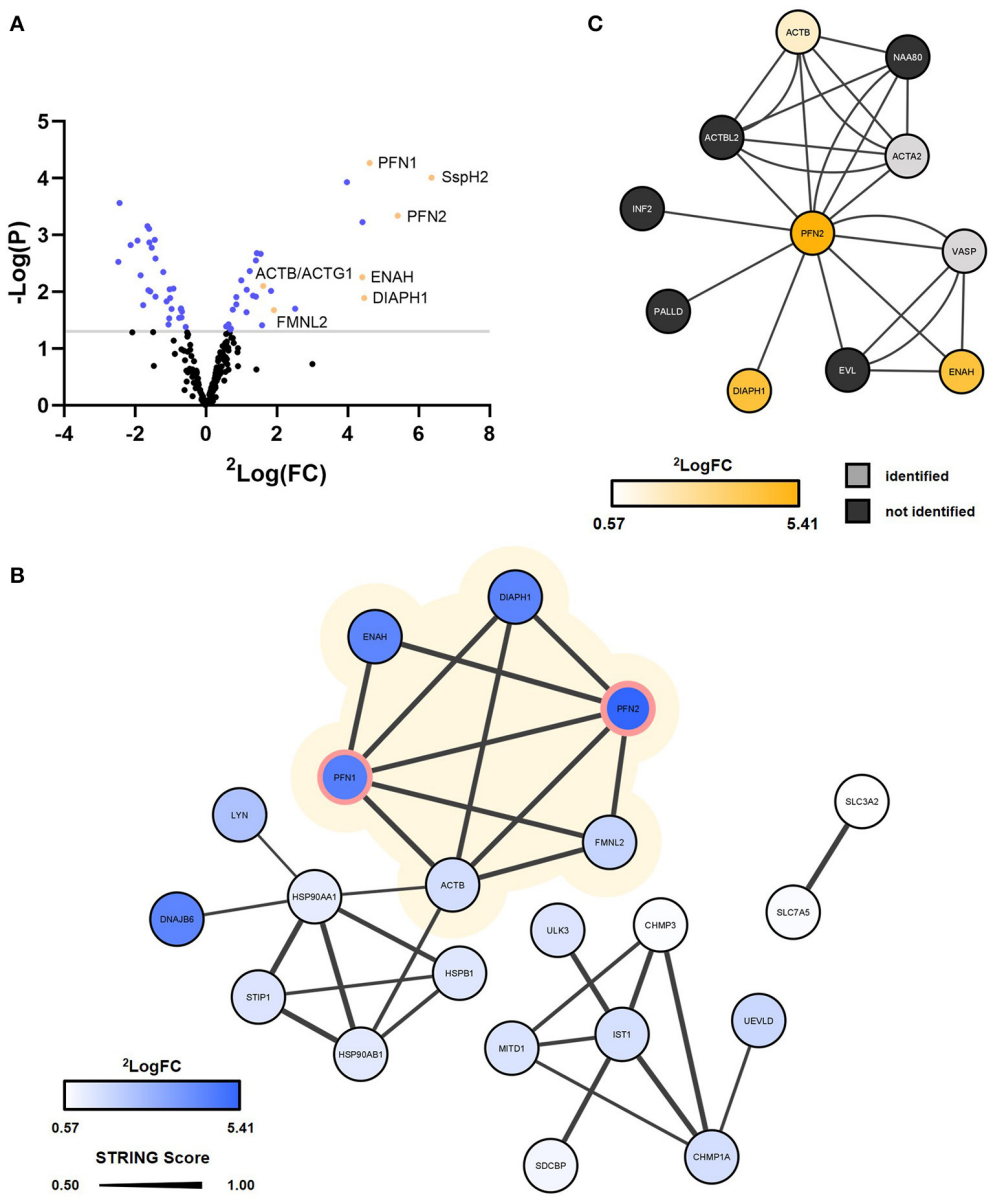
Virotrap confirms interaction of *S*. Typhimurium effector SspH2 with PFN1/2 and potential interaction with other associated actin cytoskeleton components. *S*. Typhimurium (SL1344) effector SspH2 was subjected to MS-based Virotrap analysis. **(A)** Significant hits (Limma, *p*-value < 0.05) are shown in blue and yellow [i.e., for hits defined as “actin-binding” (keyword) or actin, i.e., ACTB]. Proteins uniquely identified in the Virotrap control samples were excluded from the statistical analysis and are not shown in the volcano plot. **(B)** Gag-SspH2 co-enriched proteins (Limma, *p*-value < 0.05) are visualized in a STRING interaction network with the known interactors of SspH2 (PFN1 and−2) lined in pink (Table 1). The protein SspH2 interaction network associated with actin (-binding) is shaded in yellow. Significantly enriched proteins disconnected from the networks were not included in the network representation. **(C)** The BioPlex 3.0 interaction network characterized using C-terminally FLAG-HA-tagged PFN2 by means of anti-HA affinity purification (AP)-MS in HEK293T. Corresponding log2 fold changes of GAG-SspH2 co-enriched proteins are color-coded in the network nodes. VASP did not meet the selection criteria for quantification and ACTA2 was not regulated in the Virotrap dataset.

To enable trapping of *S*. Typhimurium SspH2 host protein interactors within VLPs, Gag-fusion proteins were expressed in HEK293T cells. More specifically, Gag-fusions of SspH2 and *Escherichia coli* dihydrofolate reductase protein (eDHFR), a non-specific bait serving as a control reference, were expressed along with (FLAG-tagged) vesicular stomatitis virus glycoprotein (VSV-G) (Figure 2), the latter exposed at the surface of the VLPs produced, enabling immunoprecipitation of VLPs. To validate VLP formation when expressing the effector fusion, anti-FLAG purification of the VLPs from the cell medium of Gag-SspH2 expressing cells was performed. As observed in 3 replicate analyses, immunoblotting against Gag revealed the potential of Gag-SspH2 to yield VLPs (Figure 3A). Further, VLPs of effector and control setups were collected, VLP protein extracts generated and tryptic digests subjected to LC-MS/MS. To determine Gag-SspH2 co-enriched proteins, label-free quantification (LFQ) was performed using the MaxLFQ algorithm (34). Membrane (-associated) proteins amounted to 54% of the identified proteins in the total Virotrap dataset, an observation in line with previous Virotrap studies (35) and indicating the ability of Virotrap to identify this protein category. Out of the total of 620 unique protein identifications in the complete Virotrap dataset (Supplementary Table 1), 231 proteins were identified in both SspH2 replicates (i.e., Virotrap enriched and background proteins) and used for relative quantification with the Gag-eDHFR setup in Limma. After log2 transformation of LFQ intensities and imputation of missing values, Pearson correlations of 0.962 and 0.972 were observed among replicate samples expressing Gag-SspH2 and Gag-eDHFR, respectively, indicating a high replicate reproducibility (Figure 4A). Pairwise analysis (Limma) using LFQ intensities of the SspH2 and control setup revealed 28 significantly enriched proteins (*p*-value < 0.05) in the Gag-SspH2 setup (Figures 4B, 5; Supplementary Tables 1, 2), including the effector fusion itself (i.e., Gag-SspH2). Consistent with previous reports (36), proteins enriched in the Gag-eDHFR control samples (e.g., TRIM32) were identified in our control setup (data not shown). As reported previously (22), other characteristic Virotrap background proteins, i.e., known Gag interactors, include endosomal sorting complexes required for transport III (ESCRT-III) proteins, such as the charged multivesicular body proteins CHMP3 and CHMP1A (implicated in vesicle extrusion and thus VLP formation), and should thus be handled with caution when identified as significantly enriched in bait samples.

**Table 1:** Overview of literature confirmed SspH2 interactors.

**Host protein**	**Model system**	**Info**	**Tag orientation**	**Method**	**Publication**
SUGT1	HEK293T cells	Overexpression bait	N-terminal (HA)	SILAC/AP-MS	(14)
		Overexpression bait	N-terminal (HA)	co-IP	
	HeLa cells	Infection context	N-terminal (HA)	SILAC/AP-MS	(15)
NOD1	HeLa cells	Overexpression bait and prey	bait N-terminal (HA), prey C-terminal (FLAG)	co-IP	(9)
PFN1	Yeast	Ectopical expression bait and prey	bait N-terminal (Gal4BD), prey N-terminal (Gal4AD)	Y2H	(16)
	HEK293T cells	Overexpression bait	N-terminal (GST)	co-IP	
	HeLa cells	Infection context	N-terminal (HA)	SILAC/AP-MS	(15)
	HEK293T cells	Overexpression bait	N-terminal (Gag)	Virotrap	This study
PFN2	HeLa cells	Infection context	N-terminal (HA)	SILAC/AP-MS	(15)
	HEK293T cells	Overexpression bait	N-terminal (Gag)	Virotrap	This study
FLNA	Yeast	Ectopical expression bait and prey	bait N-terminal (Gal4BD), prey N-terminal (Gal4AD)	Y2H	(16)
	HeLa cells	Infection context	N-terminal (HA)	SILAC/AP-MS	(15)

*HEK293T, human embryonic kidney 293T; HA, Hemagglutinin; SILAC, stable isotope labeling with amino acids in cell culture; AP-MS, affinity purification mass spectrometry; co-IP, co-immunoprecipitation; Y2H, yeast-two-hybrid*.

### Virotrap Confirms Interaction of SspH2 With PFN1 and −2

It is well-established that SspH2 co-localizes with the actin cytoskeleton and more specifically with polymerizing actin (7, 16, 37). Statistical analysis of the Virotrap data revealed co-enriched proteins with Gag-SspH2 compared to the Virotrap control setup using Gag-eDHFR (Figure 5A; Supplementary Table 2). Following protein network analysis, an actin (-binding) network cluster could be detected among the significantly enriched candidate hits for Gag-SspH2, constituting of actin gamma 1/actin beta (ACTG1/ACTB), diaphanous related formin 1 (DIAPH1), enabled homolog (ENAH), profilin-1 (PFN1), PFN2 and formin-like 2 (FMNL2) (Figure 5B; Supplementary Table 2). These data confirm previous reported results using yeast-2-hybrid (Y2H), GST-pulldown and AP-MS reporting PFN1 and−2 as (direct) host interactors of SspH2 (15, 16), and was further confirmed here for the identified Gag-SspH2/PFN1 interaction using reciprocal co-IP (Figure 3B). Profilins are known for directing actin monomers to the barbed, or fast-growing, ends of actin filaments (38) and this by interacting with formins (39) and Mena/VASP proteins (40). Remarkably, two formins (formin homology proteins), DIAPH1 and FMNL2, and the Mena/VASP protein ENAH were observed among the actin-binding proteins significantly enriched in the SspH2 Virotrap setup. Of note, DIAPH1, ENAH and ACTB have previously also been identified as interaction partners of PFN2 in HEK293T by means of AP-MS (Figure 5C) (41). In line with the observations made by Miao and colleagues, SspH2 interaction with actin-binding formins and ENAH proteins suggest the specific localization of SspH2 at sites of actin polymerization.

### Identification of NLR Signaling Chaperones as SspH2 Interactors Using Virotrap

More recently, the host protein Suppressor of G2 allele of SKP1 homolog (SUGT1) was identified as another binding partner of SspH2 (14). A follow-up study characterized this interaction and showed a role for SspH2 in enhancing NOD1 signaling in a SUGT1-dependent manner (9). In addition, SUGT1 was found to stabilize the active conformation of the SspH2 E3 ubiquitin ligase, required for NOD1 ubiquitination by SspH2. While SUGT1 was uniquely identified by MS/MS only in one of the Virotrap Gag-SspH2 samples, the two SUGT1 co-chaperones HSP90AA1 and -AB1 were identified as co-enriched with Gag-SspH2 (Figure 5B). NOD1 however, was not identified in Virotrap, an observation in agreement with its reported low abundance in (HEK293T) cell lines (Human Protein Atlas available from http://www.proteinatlas.org).

## Discussion

In recent years, facilitated by advances in MS-based interactomics strategies such as crosslinking AP-MS and proximity-dependent biotin identification (BioID), extensive mapping of *S*. Typhimurium effectors and their host targets has started to emerge (18, 19). The viral-like particle trapping technology Virotrap was only recently introduced into the interactomics field and has demonstrated its value to successfully capture binding partners of Gag-tagged human baits and even outperform classical AP-MS in this regard (22, 35, 36). As multimerization of Gag happens in the cytosol, Virotrap baits should preferentially form complexes (for fulfilling their role) in the cytosol. In contrast to AP-MS, and by means of covalent tagging and avoiding lysis conditions, respectively, BioID and Virotrap avoid false positive and/or negative interactions following the loss of cellular context. Although crosslinking can alleviate the major shortcoming of not identifying hydrophobic or weak interactors in AP-MS, it provides a momentary view on the proximate interactome (42). This is in stark contrast to the interactome retrieved by Virotrap and BioID that includes the cellular and dynamic context of the protein under study and allows for identification of interacting (and proximate) proteins irrespective of the strength of interaction (22, 43). While BioID is limited to identifying targets presenting an accessible and free Lys residue to the bait, certain Gag-bait fusions may be hindered in VLP formation in Virotrap. Both state-of-the-art interactomics platforms represent a complementary means and may well-provide, depending on its specific biology, an extensive interactome for an effector protein of interest (44). Notably, a bias toward identifying SPI-2 effector targets during infection conditions—attributed to their timing of secretion and abundancy during infection—became apparent in the study by Walch and coworkers, and evident from the selection of five SPI-2 effectors for BioID interaction screening during infection (18, 19). Virotrap might thus prove to be especially valuable in supplementing the *S*. Typhimurium effector-host interactome for the less characterized SPI-1 effectors. We here laid out a proof-of-concept study for identifying EH-PCs using Virotrap. With its multiple described host interactors and published crystal structure, we deemed the *S*. Typhimurium NEL E3 ligase SspH2 a suitable candidate to illustrate the utility and applicability of Virotrap as a proof-of-concept for studying effector-host interactions.

Using Virotrap, 620 unique proteins were identified after MS-based analysis and about half of the proteins identified in the complete dataset were annotated (GOCC) as membrane-embedded or -associated, which might offer an interesting feature, surely when considering membrane-attached baits like *S*-palmitoylated SspH2. Among the identified proteins, 27 proteins appeared to be co-enriched with Gag-SspH2 with an average fold enrichment of 7.4, indicating a putative interaction of these hits with SspH2 (Figures 4B, 5; Supplementary Table 2). Importantly, substantial care should be taken with proteins from the ESCRT-III machinery identified as enriched. As VLP formation can be variable among Gag fusions, proteins implicated in vesicle budding may appear differentially regulated in bait samples. Consequently, the validity of the identified Gag-SspH2 enriched sub-network including CHMP3 and CHMP1A (Figure 5B) may be considered questionable and was not further considered here.

In line with previous studies (15, 16), MS-based Virotrap analysis enabled identification of PFN1 and−2 (61.4% amino acid sequence identity) as binding partners of SspH2 (Figure 5). Additionally, we could confirm the Gag-SspH2 interaction with PFN1 using co-IP of VLP producer cell lysates (Figure 3B). Of note, although probing of interactors captured in VLPs using Western blot has been successfully done before (22), this proved to be challenging due to the generally low amount of VLP material (data not shown). Direct interaction of profilin and SspH2 is assumed as this interaction was also identified by means of Y2H screening (16). Confirmation of these well-characterized SspH2 interactors implies that Virotrap is indeed capable of capturing host interactors of the effector bait under study. Other significantly Gag-SspH2 co-enriched proteins include additional components of the actin cytoskeleton, which is consistent with the described localization of SspH2 at the apical membrane cytoskeleton and at VAP (16). Similar results were observed by Fiskin and coworkers in a quantitative SILAC-based AP-MS study using hemagglutinin (HA)-SspH2 as bait in HeLa cells, which resulted in the identification of several components of the actin cytoskeleton, including PFN1 and −2, FLNA and -B and alpha-actinin-4 (ACTN4) (15) as interactors. While these previous studies delivered a clear overlap of the SspH2-host protein complexes (Table 1) (15, 16), Walch and co-workers did not acquire significantly enriched hits for a natively delivered C-terminally tagged SspH2 in HeLa cells. Nonetheless, they did acquire significant hits in RAW264.7 cells using this strategy, including host protein MYH9 also found by Fiskin and coworkers using N-terminal tagged SspH2, suggesting at least a partially overlapping interactome profile of both tagged SspH2 forms (19). It remains to be investigated whether orientation of tagging or timing of pull-down during infection among others explain the absence of the described interactors of SspH2 in the Walch study.

**Figure 6:**
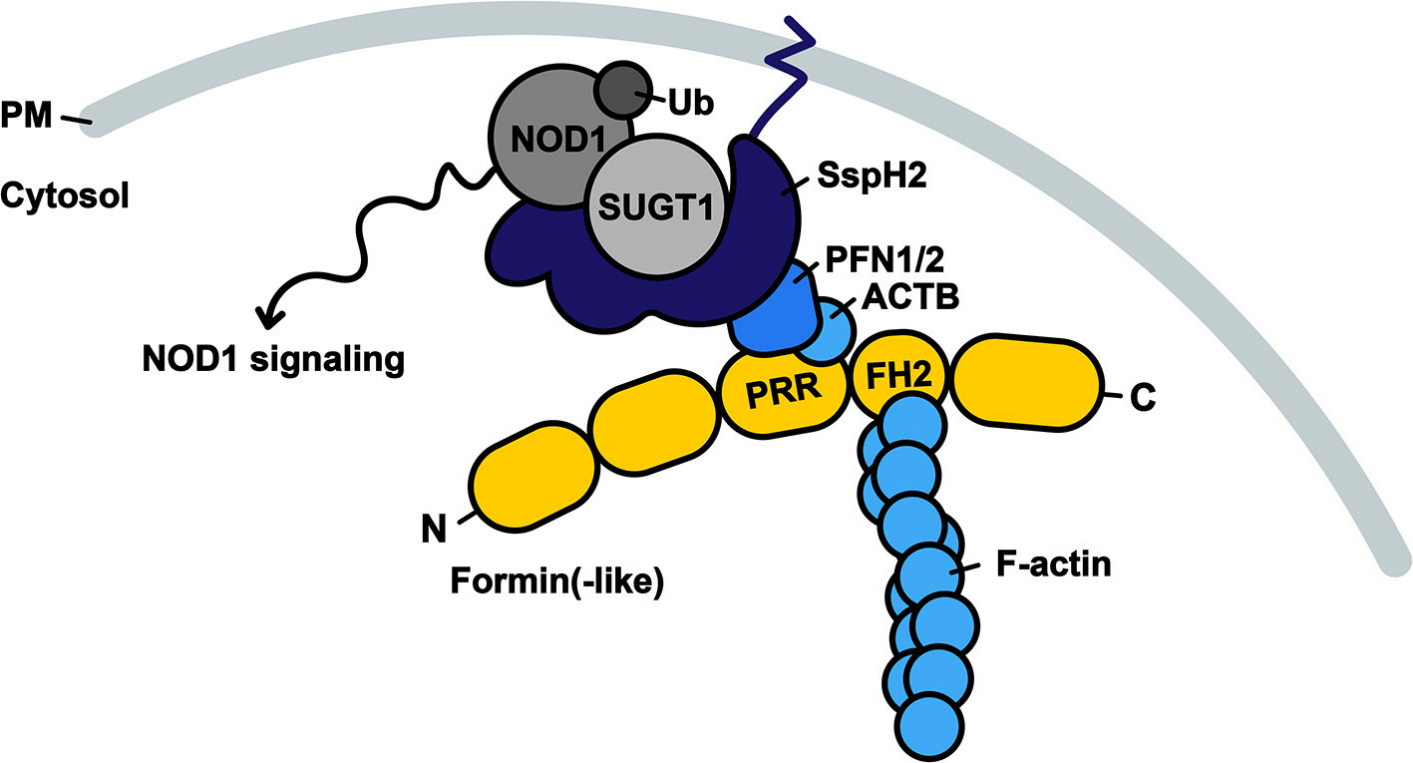
Model of SspH2 resulting in SUGT1-dependent NOD1 signaling at the dynamic actin cytoskeleton. SspH2 is localized to the plasma membrane (PM) by *S*-palmitoylation (dark blue zigzag line). Subsequent interaction with profilins (PFN1 and −2) specifies the localization of effector SspH2 further toward polymerizing actin filaments as a result of profilin binding to proline-rich regions (PRRs) of formin(-like) proteins (yellow), such as DIAPH1 and FMNL2. Barbed ends of actin filaments are bound to formins through formin-homology domains (FH2). SspH2 localized at the actin cytoskeleton allows for NOD1 signaling upon SUGT1 binding and E3 ubiquitin ligase activation. Proteins in greyscale represent proteins that were not (significantly) enriched as SspH2 interactors using Virotrap. Interactors in shades of blue were previously reported and significantly enriched in the Gag-SspH2 Virotrap samples.

SspH2 and SseI have a substantial amino acid sequence identity in the first 129 amino acids. Consequently, their shared co-localization at the actin cytoskeleton was first proposed to be mediated by interaction of their N-terminus with FLNA as inferred from Y2H studies (16). Later on, both SspH2 and SseI were uncovered as being *S-*palmitoylated at Cys9, which supports their plasma membrane localization (17). Nonetheless, *S-*palmitoylation alone did not explain why SspH2 and SseI ultimately accumulated at distinct locations, i.e., SspH2 localized to the apical membrane in microvilli and SseI mainly localized to the basolateral membrane in polarized epithelial cells (7, 17). Based on experiments using truncated effector variants, it was further speculated that additional determinants residing in the C-terminal part guide effector localization of both effectors. Interestingly, one report shows direct binding and co-localization of SseI with host factor IQ motif containing GTPase activating protein 1 (IQGAP1) (45), a scaffolding protein that localizes to the basolateral membranes and accumulates at sites of actin polymerization (46). In line with these observations, we hypothesize an alternative mode of cytoskeleton association, namely through binding of formins (DIAPH1 and FMNL2) and Mena/VASP proteins (ENAH) that bind among others profilins and growing actin filaments through their proline-rich repeat (PRR) and formin-homology 2 (FH2) domain, respectively (Figure 6). The SspH2/PFN interaction was confined to the C-terminal portion (NEL) of the effector, consistent with the hypothesis that the effector C-terminus further specifies the localization pattern (17). Profilin promotes ADP/ATP exchange upon interaction with globular actin, such as ACTB, and thereby enhances actin filament polymerization while bound to formins or Mena/VASP proteins. Since formins and Mena/VASP proteins are thus found at sites of actin polymerization, these observations coincide with the described cellular localization of SspH2 (16). Further validations and studies of these putative novel interactions should yield conclusive answers to the mechanism of SspH2 actin association.

Next to *S*. Typhimurium effectors SipA and SopE, SspH2 was found to activate NOD signaling (9, 47, 48). On the condition that SspH2 ubiquitin ligase activity was functional and NLR co-chaperone SUGT1 was bound, enhanced NOD1-mediated IL-8 secretion was observed in HeLa cells upon overexpression of the effector (9). As SUGT1 binding to SspH2 showed to increase ubiquitination *in vitro*, it was speculated that binding of the co-chaperone stabilizes the active conformation of SspH2, thereby permitting NOD1 monoubiquitination. Our data was not conclusive for the proclaimed interaction of SspH2 with SUGT1 (9, 14, 15), but co-chaperones HSP90AA1 and -AB1 were nonetheless significantly enriched in the Gag-SspH2 samples. As NOD1 is amongst the lowest expressed proteins reported in available cell lines (Human Protein Atlas available from http://www.proteinatlas.org), including HEK293T, it is not surprising that we could not identify NOD1 in Virotrap. Notably, we were also not able to detect NOD1 in our control HEK293T shotgun proteome analysis performed (~5,000 proteins identified, data not shown).

Although SspH2 was shown to decrease actin polymerization *in vitro*, cells infected with an *sspH2* deletion mutant of *S*. Typhimurium did not exhibit altered VAP compared to cells infected with the WT strain (16), therefore rendering the direct (and sole) effect of SspH2 on actin dynamics debatable. Rather, it can be postulated that localization of SspH2 at the actin cytoskeleton serves to bring the E3 ligase to its site of action for manipulation of immune signaling, i.e., near NOD1 (Figure 6). It has become increasingly clear that the cytoskeleton provides a platform for integration of immune signaling (49–51) and NOD1 is known to reside at the plasma membrane and co-localize with F-actin (52). Hence, we hypothesize that binding of SspH2 with profilin mediates its subcellular localization specifically to sites enriched with NOD1, which is in accordance with the actin cytoskeleton co-enrichments (in Virotrap) and localization of SspH2 reported (7, 16, 17).

Taken together, Virotrap may thus serve as a platform for the mapping of EH-PCs. Virotrap enabled us to verify and extend the *S*. Typhimurium SspH2 host interactome, yielding novel insights on NOD1 signaling integration at the actin cytoskeleton. Further research is deemed necessary to validate the novel SspH2 candidate targets identified, and to characterize the function of monoubiquitination of the NLR at the actin cytoskeleton and its implications in immune signaling during *S*. Typhimurium pathogenesis.

## Data Availability Statement

The datasets presented in this study can be found in online repositories. The names of the repository/repositories and accession number(s) can be found below: PRIDE database (http://www.ebi.ac.uk/pride) with the dataset identifier PXD026740 (Project name: Capturing Salmonella SspH2 Host Targets in Virus-Like Particles).

## Author Contributions

PVD: experimental design and funding acquisition. MDM and VJ: experimental execution. MDM, IF, and PVD: data analysis. MDM and PVD: writing—original draft preparation. MDM, IF, SE, and PVD: writing—review and editing. MDM: visualization. SE and PVD: supervision. All authors have read and agreed to the published version of the manuscript.

## Funding

This research was funded by the European Research Council (ERC) under the European Union's Horizon 2020 research and innovation program (PROPHECY grant agreement No 803972).

## Conflict of Interest

The authors declare that the research was conducted in the absence of any commercial or financial relationships that could be construed as a potential conflict of interest.

## Publisher's Note

All claims expressed in this article are solely those of the authors and do not necessarily represent those of their affiliated organizations, or those of the publisher, the editors and the reviewers. Any product that may be evaluated in this article, or claim that may be made by its manufacturer, is not guaranteed or endorsed by the publisher.

## Supplementary Material

Supplementary Table 1 Virotrap protein identifications after removal of contaminants, reversed hits and identifications only made by site.

Supplementary Table 2 Virotrap Gag-SspH2 co-enriched significant hits (p<0.05) using Limma.
